# Can we predict patients that will not benefit from invasive mechanical ventilation? A novel scoring system in intensive care: the IMV Mortality Prediction Score (IMPRES)

**DOI:** 10.3906/sag-1904-96

**Published:** 2019-12-16

**Authors:** Tevfik ÖZLÜ, Mehtap PEHLİVANLAR KÜÇÜK*, Akın KAYA, Esra YARAR, Cenk KIRAKLI, Özlem ŞENGÖREN DİKİŞ, Hale KEFELİ ÇELİK, Serdar ÖZKAN, Hayriye BEKTAŞ AKSOY, Ahmet Oğuzhan KÜÇÜK, IMVICAP Study Group

**Affiliations:** 1 Department of Chest Diseases, Faculty of Medicine, Karadeniz Technical University, Trabzon Turkey; 2 Department of Chest Diseases, Division of Intensive Care Medicine, Faculty of Medicine, Karadeniz Technical University, Trabzon Turkey; 3 Department of Chest Diseases, Faculty of Medicine, Ankara University, Ankara Turkey; 4 Department of Chest Diseases, Necip Fazıl City Hospital, Kahramanmaraş Turkey; 5 Department of Chest Diseases, İzmir Dr Suat Seren Thoracic Diseases and Surgery Training and Research Hospital, İzmir Turkey; 6 Department of Chest Diseases, Bursa Yüksek İhtisas Training and Research Hospital, University of Health Sciences, Bursa Turkey; 7 Department of Anesthesiology and Reanimation, Samsun Training and Research Hospital, Samsun Turkey; 8 Department of Thoracic Surgery, Faculty of Medicine, Karatay University, Konya Turkey; 9 Department of Chest Diseases, Prof. Dr. A. İlhan Özdemir Training and Research Hospital, Giresun University, Giresun Turkey; 10 Department of Anesthesiology and Reanimation, Division of Intensive Care Medicine,Faculty of Medicine, Karadeniz Technical University, Trabzon Turkey

**Keywords:** Critical care, ethics, intubation, invasive mechanical ventilation, scoring systems, prediction

## Abstract

**Background/aim:**

The present study aimed to define the clinical and laboratory criteria for predicting patients that will not benefit from invasive mechanical ventilation (IMV) treatment and determine the prediction of mortality and prognosis of these critical ill patients.

**Materials and methods:**

The study was designed as an observational, multicenter, prospective, and cross-sectional clinical study. It was conducted by 75 researchers at 41 centers in intensive care units (ICUs) located in various geographical areas of Turkey. It included a total of 1463 ICU patients who were receiving invasive mechanical ventilation (IMV) treatment. A total of 158 parameters were examined via logistic regression analysis to identify independent risk factors for mortality; using these data, the IMV Mortality Prediction Score (IMPRES) scoring system was developed.

**Results:**

The following cut-off scores were used to indicate mortality risk: <2, low risk; 2–5, moderate risk; 5.1–8, high risk; >8, very high risk. There was a 26.8% mortality rate among the 254 patients who had a total IMPRES score of lower than 2. The mortality rate was 93.3% for patients with total IMPRES scores of greater than 8 (P < 0.001).

**Conclusion:**

The present study included a large number of patients from various geographical areas of the country who were admitted to various types of ICUs, had diverse diagnoses and comorbidities, were intubated with various indications in either urgent or elective settings, and were followed by physicians from various specialties. Therefore, our data are more general and can be applied to a broader population. This study devised a new scoring system for decision-making for critically ill patients as to whether they need to be intubated or not and presents a rapid and accurate prediction of mortality and prognosis prior to ICU admission using simple clinical data.

## 1. Introduction

Mortality rates in adult ICUs range from 30% to 65% [1–5]. Invasive mechanical ventilation (IMV) is often necessary during the course of serious acute pathologies such as traumas, intoxications, and infections, as well as during the course of chronic diseases such as neuromuscular disorders, chronic obstructive pulmonary disease (COPD), and interstitial lung diseases [6–8]. The criteria for intensive care unit (ICU) admission and discharge as well as indications for IMV treatment have been established [9,10]. However, not all patients undergoing IMV benefit from this treatment. For such cases, IMV only helps to postpone mortality. The suspended animation state that will inevitably result in death is often spent in a sedated, comatose, and completely passive condition with a very low quality of life, which can be quite tormenting for the patient. Since patients requiring IMV are often admitted to the ICU, a significant number of the limited beds in ICUs are often occupied by patients who will not survive. Because of this, many patients with reversible conditions requiring ICU care will not have access to ICU support. Moreover, comatose patients receiving IMV can make it difficult to control serious problems in the ICU, such as nosocomial infections. Further, the relatives of these patients often have irrational hopes for recovery, which leads to prolonged IMV treatment. Many countries commonly practice the orders “do not resuscitate” (DNR) and “do not intubate” (DNI), meaning that either the patient or his/her custodian had decided to forego life-prolonging treatment when resuscitation is not expected to change the survival outcome [11]. For instance, in Taiwan, if a patient older than the age of 20 provides a written statement acknowledging his/her will to abandon medical treatment, then according to the Natural Death Act (passed in the year 2000), that patient’s doctor is not subject to legal sanction. However, DNR documents and other advance orders are not yet part of standard medical practices in low-income countries [12]. 

In this regard, the accurate prediction of patients that will most likely benefit from IMV is very important for clinicians when justifying IMV in emergency departments, clinics, or, sometimes at the scene of the event. Unfortunately, to our knowledge, there are no evidence-based and tangible criteria used for defining patients that will not benefit from IMV. Such criteria would save clinicians from ethical dilemmas and protect them in judicial processes.

In the current study, we aimed to define criteria that will help to objectively identify patients that will not benefit from IMV treatment. With these criteria, we hope to eliminate the impact of subjective personal anticipation, the insistence and pressures of patients’ relatives, and local-cultural determinants. We hope that this study will serve to ease clinicians’ decision-making processes in the face of jurisdiction, patients’ relatives, and their own conscience, as well as aiding in objective decision-making and facilitating the more rational use of ICU beds.

## 2. Materials and methods

### 2.1. Study design

The STROBE guidelines were used as a guide for this manuscript. This study was designed as an observational, multicenter, prospective, and cross-sectional clinical study. The scientific ethical committee of Karadeniz Technical University’s Faculty of Medicine granted ethical approval for this study. Patients/custodians and researchers provided written consent prior to participation in this study. Researchers from various ICUs in Turkey who accepted the invitation that was distributed nationwide (via e-mail) were enrolled in the study. An online meeting was held among the participating researchers to establish the study protocol and the data collection form. The results were evaluated in an e-mail group that included all of the researchers, and the current manuscript was composed in accordance with the opinions and recommendations of all of the researchers.

### 2.2. Patients and setting

This study included patients who were receiving IMV treatment in ICUs. This was completely an observational study, and no extra interventions were applied to the patients. Bedside data collection forms that were created specifically for this study were filled out by researchers for all patients who stayed in the ICU for more than 24 h and received IMV support. At the end of each month, data from patients who were discharged from the ICU within that month were collected from every center. Patients who died during their ICU stay were categorized as the group that did not benefit from IMV, while the surviving patients (e.g., transferred to a ward, discharged to home, referred, etc.) were categorized as the group that benefited from IMV. This study excluded patients who were admitted to the pediatric ICU, neonatal ICU, or postanesthesia care units and those who were younger than 18 years old. For every patient, we collected demographical data, the type of the ICU to which they were admitted, primary indications for ICU admission, comorbid diseases, place of intubation (i.e. event scene, emergency department, hospital ward, ICU, other), urgency of intubation (i.e. urgent, elective), and the physician who decided on intubation and intubation indications. Additionally, we evaluated the possible patient-related factors that were considered by the physician as an indicator that IMV would most likely not benefit a patient (Table 1) [1,13].

**Table 1:** Possible patient-related factors suggesting that the patient will most likely not benefit from IMV treatment [1,13].

Serious comorbidity (one or more)
Advanced age
Low chance of recovery despite the benefits gained
Low chance for life-prolonging treatment
Bed-bound for the long term (>3 months)
Terminal stage of chronic disease/malignancy
Life expectancy shorter than 6 months
Permanent multiorgan failure
Malignancy refractory to previous chemotherapy/radiotherapy
Recurring ICU requirement due to development of serious organ failure following discharge from previous prolonged ICU admission
High treatment cost in proportion to the benefits gained
IMV requirement in an immunosuppressed patient as a result of the primary disease
Newly diagnosed patient who is unlikely to tolerate chemotherapy treatment

For all patients, we recorded the condition at discharge (e.g., exitus, successful weaning/extubation, mechanical ventilation dependence/tracheostomy, referral), reintubation requirement, mechanical ventilation duration, and ICU stay length. On the first day of ICU admission, we calculated the Acute Physiology and Chronic Health Evaluation-II (APACHE-II) and Sequential Organ Failure Assessment (SOFA) scores for each patient [14,15].

### 2.3. Statistical analysis

All data for this study were analyzed with IBM SPSS 23 statistics software. The Shapiro–Wilk test was used to determine the normality of the numerical data. Nonnormally distributed numerical data were analyzed with the Mann–Whitney U test via nonparametric methods. Comparisons of categorical data were made with the Pearson chi-square test. Independent risk factors for mortality were determined with binary logistic regression analysis. For those risk factors found to be significant via logistic regression analysis, odds ratio (OR) values were used to calculate the IMV Mortality Prediction Score (IMPRES). The chi-square test was used to compare mortality rates among groups after risk stratification. Numerical data were expressed as medians (min–max), while categorical data were presented as frequencies (percentages). Values of P < 0.05 were considered significant.

## 3. Results

### 3.1. General patient characteristics 

The study was conducted with the participation of 75 researchers from 41 distinct centers (universities, training and research hospitals, or state hospitals) located in various geographical areas of Turkey. Data collection was performed from 1 January 2017 to 30 April 2017. A total of 1463 patients receiving IMV treatment in 11 different types of ICUs located in these centers during the study period were enrolled in this study. Of these patients, 625 (42.7%) were female and 838 (57.3%) were male. The median patient age was 71 years (18–101 years), and the median body mass index (BMI) was 26 kg/m2 (14­­–76 kg/m2). Of the patients, 639 (43.7%) were from university hospitals, 530 (36.2%) were from training and research hospitals, 220 (15%) were from state hospitals, and 74 (5.1%) were from private hospitals. Of the patients, 762 (52.1%) were followed by attending physicians other than an ICU specialist, 397 (27.2%) were followed by an ICU specialist/fellowship trainer, and 304 (20.8%) were followed by an ICU physician in-chief. The type of ICU was general ICU for 429 (29.3%) patients, anesthesiology and reanimation ICU for 268 (18.3%) patients, medical ICU for 210 (14.4%) patients, and pulmonary diseases ICU for 154 (10.5%) patients. Other ICU types included surgical ICUs, emergency departments, and neurological, neurosurgical, internal diseases, coronary, and cardiovascular ICUs. Table 2 presents the clinical features of the patients.

**Table 2:** Characteristic features of the patients included in this study.

	n (%)		n (%)
Primary indication for admission		Comorbidities	
Pneumonia	415 (28.37)	Hypertension	536 (36.64)
COPD exacerbation	247 (16.88)	Heart failure	326 (22.28)
Acute renal failure	229 (15.65)	Diabetes mellitus	306 (20.92)
Heart failure	201 (13.74)	COPD	266 (18.18)
Cardiac arrest	179 (12.24)	Coronary arterial disease	264 (18.05)
Sepsis	165 (11.28)	None	182 (12.44)
Cerebrovascular ischemia	152 10.39)	Arrhythmia	165 (11.28)
Chronic renal disease	102 (6.97)	CVA	148 (10.12)
Aspiration	96 (6.56)	Alzheimer’s disease	118 (8.07)
Cerebrovascular hemorrhage	94 (6.43)	Chronic renal failure	108 (7.38)
Pulmonary edema	92 (6.29)	Lung cancer	65 (4.44)
Hyper/hypotension	85 (5.81)	Chronic renal disease	39 (2.67)
Lung malignancy	77 (5.26)	Asthma	34 (2.32)
Arrhythmia tachy/bradycardia	73 (4.99)	Heart valvular disease	30 (2.05)
Acute coronary syndrome	62 (4.24)	Hyper/hypothyroidism	26 (1.78)
Coronary arterial disease	47 (3.21)	Colon/intestinal cancer	25 (1.71)
Post-operative (elective)	43 (2.94)	Epilepsy	25 (1.71)
Multiple trauma	42 (2.87)	Other	428 (29.25)
Pulmonary embolism	42 (2.87)	Acute indication for ICU admission	
Intracranial trauma	41 (2.80)	Type I respiratory failure	518 (35.41)
ARDS	40 (2.73)	Deteriorating GCS	438 (29.94)
Other	757 (51.74)	Type II respiratory failure	417 (28.50)
Indication for intubation		Cardiopulmonary resuscitation	283 (19.34)
Insufficient oxygenation/hypoxemia	656 (44.8)	Hypotensive shock	133 (9.09)
Orientation-cooperation disturbance	516 (35.3)	Circulatory shock	84 (5.74)
Insufficient ventilation/hypercapnia	479 (32.7)	Severe electrolyte imbalance	83 (5.67)
Respiratory arrest	316 (21.6)	Circulatory failure	76 (5.19)
Cardiac arrest	299 (20.4)	Major hemorrhage	44 (3.01)
NIV failure	208 (14.2)	Type III respiratory failure	44 (3.01)
Severe metabolic acidosis	117 (8.0)	Distributive shock	28 (1.91)
Control of pulmonary secretions	109 (7.5)	Neurogenic shock	19 (1.30)
Other	105 (7.17)	Brain death – possible donor	9 (0.62)
		Other	55 (3.76)

All indications are not shown in the table. Indications are listed in order of frequency (%). COPD: Chronic obstructive pulmonary disease, ARDS: acute respiratory distress syndrome, NIV: noninvasive ventilation, CVA: cerebrovascular accident, GCS: Glasgow Coma Score.

Of the patients, 823 (56.3%) were intubated in an urgent condition, while 640 (43.7%) were intubated in an elective condition. The most common place where intubation was performed was ICUs (797 (54.5%) patients), followed by emergency departments (393 (26.9%) patients), hospital wards (156 (10.7%) patients), event scene (57 (3.9%) patients), and other locations (60 (4.0%) patients). During their ICU stay, 197 (13.5%) patients required reintubation. With regard to patient outcomes, 880 (60.2%) patients died during their ICU stay, while the rest of the patients were discharged from the ICU with the following conditions: successful weaning/extubation (368 (25.2%) patients), mechanical ventilator dependence/tracheostomy (168 (11.5%) patients), and referral or transfer to other ICUs (47 (3.2%) patients).

When comparing the nonsurvival and survival groups, patient age was significantly higher in the mortality group (P < 0.001). However, there was no difference in mortality rate between the sexes (P = 0.161). Patient mortality was also evaluated according to the acute conditions presenting as ICU admission indications. While mortality rates were lower among patients with type II (P = 0.026) and type III respiratory failure (P < 0.001), they were significantly higher among those admitted to the ICU after successful cardiopulmonary resuscitation (P < 0.001), circulatory shock (P < 0.001), distributive shock (P = 0.016), circulatory failure (P = 0.013), and severe electrolyte imbalance (P < 0.001). The diagnosis of type I respiratory failure did not cause any significant difference in mortality (P = 0.165). In terms of intubation indications, mortality was seen in 219 (73.2%) of 299 patients intubated after cardiac arrest (P < 0.001) and in 106 (51%) of 208 patients intubated after a failed attempt at noninvasive ventilation (NIV) (P = 0.003). The mortality rate did not differ according to whether the intubation was performed in an urgent or elective condition. Mortality was seen in 500 (60.8%) of 823 patients intubated in urgent settings and in 380 (59.4%) of 640 patients intubated in elective settings (P = 0.401). Characteristic properties of patients in the nonsurvival and survival groups are presented in Table 3.

**Table 3:** Characteristic properties that were significantly different between the nonsurvival and survival groups of patients receiving IMV treatment.

Variable		Total (n = 1463)	Non-survived (n = 880)	Survived (n = 583)	P-value
Demographics					
Age (years), median		71 (18–101)	73 (18–101)	69 (18–95)	<0.001
Height [10cm], median		168 (100–190)	168 (110–190)	170 (100–190)	0.034
Weight (kg), median		75 (32–160)	75 (32–160)	75 (35–149.5)	0.009
BMI, median		26 (14–76)	26 (14–76)	26 (14–60)	0.046
Indication for admission					
Thoracic trauma	No	1440	872 (60.6)	568 (39.4)	0.017
	Yes	23	8 (34.8)	15 (65.2)	
Multiple trauma	No	1421	864 (60.8)	557 (39.2)	0.003
	Yes	42	16 (38.1)	26 (61.9)	
Cardiac arrest	No	1284	755 (58.8)	529 (41.2)	0.005
	Yes	179	125 (69.8)	54 (30.2)	
Pulmonary edema	No	1371	835 (60.9)	536 (39.1)	0.023
	Yes	92	45 (48.9)	47 (51.1)	
COPD exacerbation	No	1216	758 (62.3)	458 (37.7)	<0.001
	Yes	247	122 (49.4)	125 (50.6)	
Pulmonary hypertension	No	1436	855 (59.5)	581 (40.5)	<0.001
	Yes	27	25 (92.6)	2 (7.4)	
Pulmonary malignancy	No	1386	814 (58.7)	572 (41.3)	<0.001
	Yes	77	66 (85.7)	11 (14.3)	
Acute renal failure	No	1234	711 (57.6)	523 (42.4)	<0.001
	Yes	229	169 (73.8)	60 (26.2)	
Chronic renal failure	No	1361	809 (59.4)	552 (40.6)	0.043
	Yes	102	71 (69.6)	31 (30.4)	
Sepsis	No	1298	752 (57.9)	546 (42.1)	<0.001
	Yes	165	128 (77.6)	37 (22.4)	
Neurodegenerative disease	No	1437	873 (60.8)	564 (39.2)	<0.001
	Yes	26	7 (26.9)	19 (73.1)	
Oncological solid tumor	No	1437	857 (59.6)	580 (40.4)	<0.001
	Yes	26	23 (88.5)	3 (11.5)	
ICU-level nursing care requirement	No	1437	855 (59.5)	582 (40.5)	<0.001
	Yes	26	25 (96.2)	1 (3.8)	
Comorbidity					
None	No	1281	786 (61.4)	495 (38.6)	0.012
	Yes	182	94 (51.6)	88 (48.4)	
Arrhythmia	No	1298	766 (59)	532 (41)	0.013
	Yes	165	114 (69.1)	51 (30.9)	
Lung cancer	No	1398	822 (58.8)	576 (41.2)	< 0.001
	Yes	65	58 (89.2)	7 (10.8)	
Chronic renal failure	No	1355	800 (59)	555 (41)	0.002
	Yes	108	80 (74.1)	28 (25.9)	
Hematological cancer	No	1448	867 (59.9)	581 (40.1)	0.023
	Yes	15	13 (86.7)	2 (13.3)	
Hospital type					
State hospital		220	134 (60.9) a	86 (39.1)	<0.001
Teaching hospital		530	301 (56.8) b	229 (43.2)	
University hospital		639	415 (64.9) a	224 (35.1)	
Private hospital		74	30 (40.5) c	44 (59.5)	
ICU score					
SOFA, median		9 (1–32)	10 (1–32)	8 (1–28)	<0.001
APACHE, median		26 (1–67)	28 (2–53)	23 (1–67)	<0.001
End point					
MV duration, median		7 (1–369)	6 (1–121)	8 (1–369)	<0.001
ICU duration, median		10 (1–369)	8.5 (1–122)	13 (1–369)	<0.001

Data are given as n (%) unless otherwise indicated. Data are presented as median (minimum–maximum) unless otherwise indicated. a, b, c: Binary chi-square test results indicate statistical differences. P < 0.05 indicates a statistically significant difference. BMI: Body mass index, COPD: chronic obstructive lung disease, APACHE-II: Acute Physiology and Chronic Health Evaluation-II, SOFA: Sequential Organ Failure Assessment, MV: mechanical ventilation, ICU: intensive care unit.

### 3.2. A novel scoring system for the prediction of ICU mortality: IMV Mortality Prediction Score (IMPRES)

A total of 158 parameters were examined via logistic regression analysis with the backward Wald method. The variables that were identified as independent risk factors for mortality are listed in Table 4. The OR values for every parameter were used to develop the IMPRES. Since not all of the parameters would have the same effect on mortality, we utilized the OR values calculated with the logistic regression analysis in this model, as these values are the best statistics for representing this difference. Logistic regression analysis identified the following independent risk factors: age, pulmonary edema, COPD exacerbation, interstitial lung disease, acute renal failure, sepsis, metabolic encephalopathy, neurodegenerative disease, ICU-level nursing care requirement, type III respiratory failure, heart failure, lung cancer, cardiac arrest, and conditions suggesting to the physician that IMV is unlikely to benefit the patient (e.g., no chance of life-prolonging treatment, serious comorbidity (one or more), life expectancy shorter than 6 months, permanent multiorgan failure, low chance of recovery despite the benefits gained, high treatment cost in proportion to the benefits gained, terminal stage chronic disease/malignancy). While ‘ICU-level nursing care requirement’ and ‘interstitial lung disease’ had the greatest effects on mortality (increasing mortality risk by 16.7 and 11.9 times, respectively), the presence of ‘COPD exacerbation’, ‘pulmonary edema’, and ‘heart failure’ had negative impacts on the score (–0.6-fold, –0.5-fold, and –0.7-fold, respectively). The scoring for each parameter is presented in Table 4.

**Table 4:** IMV Mortality Prediction Score (IMPRES).

Parameter	Points
Demographics	
Age 70 years or older	1.6
Primary indication for admission	
Pulmonary edema	–0.5
COPD	–0.6
Interstitial lung disease	11.9
Acute renal failure	1.7
Sepsis	2.2
Metabolic encephalopathy	–0.3
Neurodegenerative diseases	–0.2
ICU-level nursing care requirement	16.7
Acute indication for ICU admission	
Type III respiratory failure	–0.3
Comorbidities	
Heart failure	–0.7
Lung cancer	3.7
Indication for intubation	
Cardiac arrest	1.9
Feature suggesting that MVI is unlikely to benefit	
Lack of life-prolonging treatment chance	2.3
Serious comorbidity (one or more)	2.3
Life expectancy shorter than 6 months	3.0
Permanent multiorgan failure	2.4
Low chance of recovery despite the benefits gained	1.9
High treatment cost in proportion to the benefits gained	–0.3
Terminal stage chronic disease/malignancy	2.8

<2: Low risk, 2–5: moderate risk, 5.1–8: high risk, >8: very high risk. COPD: Chronic obstructive pulmonary disease, ICU: intensive care unit.

The total score for each patient was calculated using the OR values of the independent risk factors that were significant via logistic regression analysis. Cut-off points were determined for the total score. To determine the cut-off points, two initial categories were formed via receiver operating characteristic (ROC) analysis. Consequently, an ordinal structure was applied to further categorize the groups as low, moderate, high, and very high risk, so that the mortality rate would increase from the low to the very high risk groups and differ between the categories. Accordingly, the following cut-off scores were obtained: <2, low risk; 2–5, moderate risk; 5.1–8, high risk; >8: very high risk.

After scoring for all of the risk factors that were found to be significant via logistic regression analysis, 1463 patients were categorized in an ordinal manner according to the cut-off scores presented above. Mortality rates were compared between these risk categories and the results are given in the Figure. 

**Figure:**
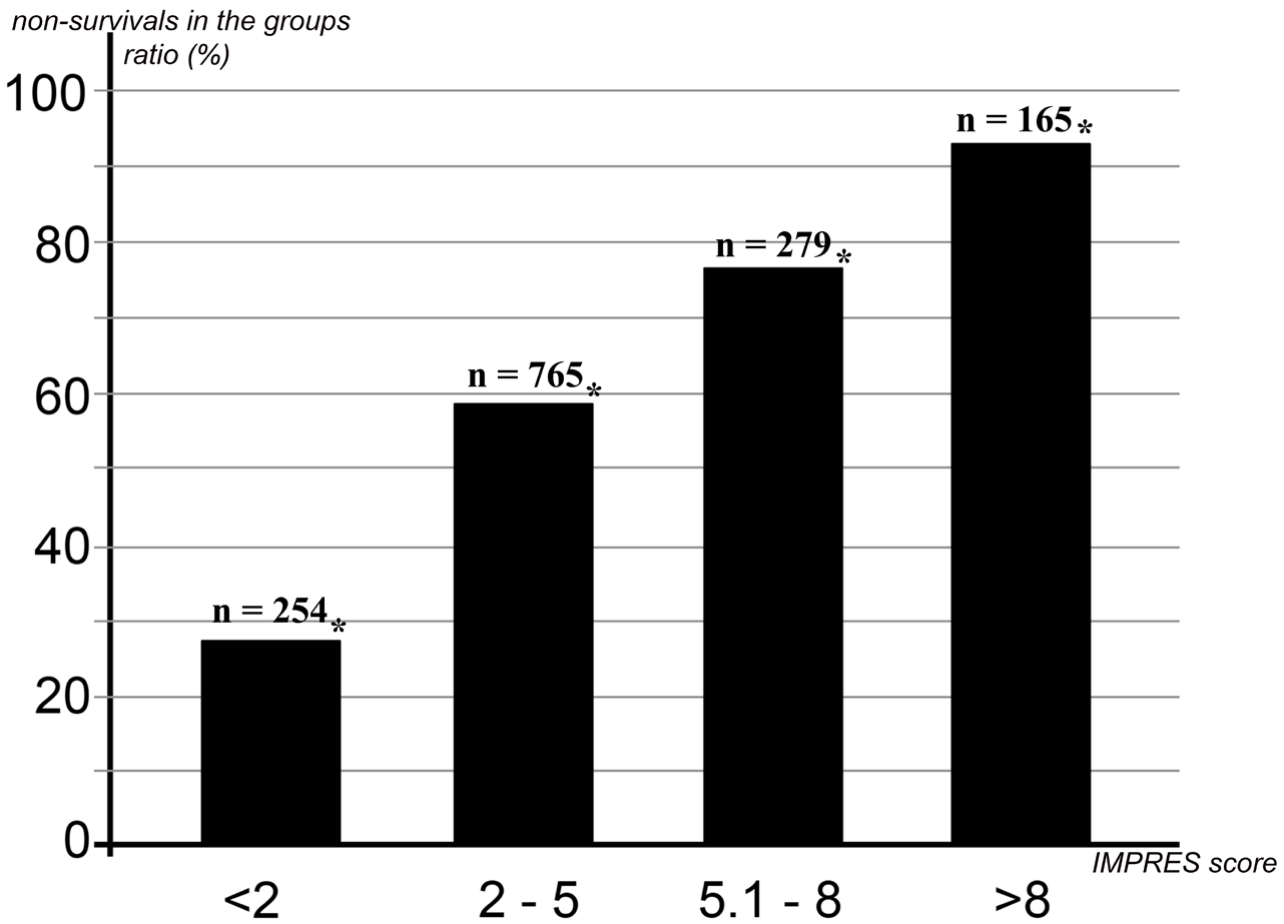
Mortality rates according to risk categories (<2: low risk, 2–5: moderate risk, 5.1–8: high risk, >8: very high risk). x-axis: IMPRES score category, y-axis: rate (%) of nonsurvival in the groups. *: Each group has a statistically significant difference in mortality rate (P < 0.001). n = total number of patients in group.

Mortality was seen in 26.8% of the 254 patients with a total score of lower than 2. The mortality rate was 58.2% among patients with a total score between 2 and 5, 76.3% among patients with a total score between 5.1 and 8, and 93.3% among patients with a total score of greater than 8. The increase in the mortality rate according to the risk categories was statistically significant (Figure).

## 4. Discussion

Physicians experience a dilemma with some patients, having to decide whether or not to initiate IMV treatment. Despite the belief that IMV will not benefit the patient, the physician may feel obligated to intubate the patient due to the insistence of the patient’s relatives, local-cultural factors, or judicial pressures. Although indications for intubation and IMV have been defined, evidence-based recommendations about patients that will not benefit from IMV and those that should not be intubated are still lacking. Therefore, the current study aimed to determine criteria that can predict which patients will not benefit from IMV. The main objective of this study was to determine a method of making rapid and accurate predictions of mortality/prognosis prior to ICU admission using simple clinical features and thus to define “priority” patients for IMV in order to facilitate the more effective use of available ICU bed capacity.

An ideal scoring system should accurately predict mortality, and the actual mortality should be close to the predicted mortality. The calculation should be convenient and be based on readily available clinical parameters without the need for advanced laboratory investigation. Scoring systems designed for the objective assessment of the clinical severity and prediction of prognosis and mortality in ICU patients are currently being used for the standardization of research and for making comparisons of the quality of care given to ICU patients. Among these, the APACHE score (I–IV) uses the worst physiological values measured within 24 h of ICU admission [16–21]. The Sequential (sepsis-related) Organ Failure Assessment (SOFA) score uses patient data within the first 24 h of ICU admission and every subsequent 48 h [22], while updated versions of the Simplified Acute Physiological Score (SAPS II–III) [23,24] and Mortality Prediction Model (MPM0 I, II, III) use data collected within the first hour of ICU admission [25–28]. These scoring systems have both advantages and disadvantages. For example, APACHE IV was developed with data collected only from hospitals in the United States and requires complex patient data. In addition, despite being developed with data collected from 35 different countries, some regional equations were developed using a relatively low sample size [24,29]. When using the existing scoring systems, clinicians should be aware of the limitations related with their unique patient populations. For instance, SAPS-III yields relatively lower mortality rates for patients with cancer or solid organ transplants, whereas SOFA can be more helpful in a population with sepsis [30–32]. The present study was unique in that it included a large number of patients from various geographical areas of Turkey who were admitted to various types of ICUs, had diverse diagnoses and comorbidities, were intubated with various indications in either urgent or elective settings, and were followed by physicians from various specialties. Therefore, we believe that our data are more general and can be applied to a broader population. Moreover, the existing scoring systems do not allow for the prediction of mortality based only on the patient’s simple clinical findings; rather, they require further laboratory investigations and 24–48 h of monitoring. However, physicians who are uncertain of whether or not to intubate require a rapid and accurate prediction of mortality based on simple clinical findings. Unfortunately, the scoring systems mentioned above do not completely satisfy this need. Indeed, we believe that our simple scoring system (IMPRES, Invasive Mechanical Ventilation Mortality Prediction Score), which was developed based on the available data, may satisfy this need. One unique feature of the IMPRES scoring system is that it also takes the physician’s anticipations and personal experiences into account in the prediction of prognosis/mortality. Rather than being a laboratory-based calculation, this scoring system prioritizes the patient’s primary diagnosis and acute needs requiring intensive care. Additionally, in the current study, the APACHE-II and SOFA scores were significantly higher in the mortality group, as expected (P < 0.001).

Many published studies have evaluated the factors associated with mortality in ICU patients. Lee et al. found that age, sex, Deyo–Charlson comorbidity index, teaching hospital, hospital level, hospital volume, and physician volume were significantly associated with mechanical ventilation outcome (P < 0.001). The ICU patient population generally consists of elderly patients. In our current study, the median age of the whole study group was 71 (18–101) years, and 67.9% of these patients were older than 65 years. One population-based cohort study from Taiwan retrospectively analyzed 213,945 patients. In this large series, all of the patients had a mechanical ventilation requirement, and 79.7% were over 65 years old [33]. One study from the United States reported that 48% of ICU patients were over 65 years old, while this rate was 38% in a study conducted in Paris [34,35]. The reason that our current study and the study from Taiwan had such high rates of elderly patients may be because these studies only included patients receiving IMV. Patients receiving IMV support are generally older because the incidence of acute respiratory failure increases significantly with every 10-year increment in age until age 85. Indeed, the incidence of acute respiratory failure in the age group of 65–84 years is 2 times higher than that of patients aged 55–64 years and 3 times higher than that of younger patients [36]. Previous studies have reported that age over 85 years is an independent factor for not being accepted to the ICU. However, there is still no global consensus regarding the admission of elderly patients (over 70–80 years) to the ICU [37]. 

Of our total study patients, 60.2% died during their ICU stay. Such a high mortality rate can be explained by the fact that this study had high average ICU APACHE-II and SOFA scores, and all of the patients included in this study had a mechanical ventilation requirement. General adult ICU mortality rates in the literature vary between 30% and 65% depending on the selected patient population [1–5]. Many previous studies have found that acute organ dysfunction is associated with short-term ICU mortality [38,39]. A review of the available data shows that there is much heterogeneity in ICU admission criteria. The heterogeneous group of patients included in the present study enabled us to examine the predictive values of many diagnoses in relation to IMV prognosis. For example, patients with pulmonary edema, COPD exacerbation, metabolic encephalopathy, and neurodegenerative diseases benefitted from mechanical ventilation. Knowing the predictive value of a patient’s primary diagnosis when deciding on IMV or ICU admission would be quite helpful for triage, or the sorting of patients considering their chance of recovery. Patients have ICU admission priority if they have severely disturbed overall conditions, are unstable, and require advanced monitoring and treatment that cannot be provided outside of the ICU. Patients with ICU admission priority include postoperative patients requiring ventilator support and treatments such as vasoactive drug infusion and patients with acute respiratory failure, hemodynamic instability, shock, severe sepsis or sepsis-septic shock, severe trauma, and hypoxia or hypotension [27,28]. There are ongoing discussions as to whether patients admitted to the ICU should have a reasonable survival expectancy and whether the patient should possess a neuropsychiatric status that is sufficient to comprehend this support. In fact, this opinion was expressed in the joint consensus statement of the Society of Critical Care Medicine [10] Ethics Committee as follows: “The primary goal of intensive care is to provide treatment to a patient with a reasonable survival expectancy beyond the acute treatment, who has adequate cognitive skills to comprehend the benefits of treatment. Intensive care interventions should be regarded as futile when there is no reasonable expectation that the patient will recover to survive beyond the acute care, or when the patient’s neurological functions are not fit to perceive the benefits of treatment” [40]. However, these recommendations are not based on any legislative regulations in Turkey, nor in many other countries. 

Physicians facing problems associated with the allocation of ICU beds for patients with low survival expectancy do not currently have the scientific evidence to aid in identifying the priority patients that they require in the face of ethics and the law. Even if a physician believes that IMV is not likely to be of any benefit to a patient, he or she may feel obliged to intubate the patient due to the lack of scientific evidence. Nevertheless, our findings may need to be verified in specialized ICUs that care for specific patient populations (e.g., hematopoietic stem cell transplant patients), or in institutions or regions where a specific disorder is prevalent (e.g., substance abuse, transplantation).

In conclusion, IMPRES takes various data into account, including the physician’s subjective anticipation of the patient’s survival. We believe that IMPRES can help physicians make a correct assessment of the patient regarding prognosis and survival at the bedside prior to deciding whether or not to intubate without requiring any further time-consuming investigations. In consideration of our heterogeneous study population, we believe that IMPRES can be used without influence arising from the type of ICU or the differences in patient populations. 

## Acknowledgments

We would like to acknowledge the Lung Health and the Intensive Care Society for their scientific support (announcement of the project to the members and promoting participation). The Lung Health and the Intensive Care Society did not provide financial support for the study. We also acknowledge the Writing Committee Members for the IMVICAP Study Group, as follows: Onur Palabıyık, Department of Anesthesiology and Reanimation, Faculty of Medicine, Sakarya University, Sakarya, Turkey; Mustafa Çörtük, Department of Chest Diseases, Faculty of Medicine, Karabük University, Karabük, Turkey; Recai Ergün, Department of Chest Diseases, Dışkapı Yıldırım Beyazıt Training and Research Hospital, Ankara, Turkey; Betül Kozanhan, Department of Anesthesiology and Reanimation, Konya Training and Research Hospital, Konya, Turkey; Özlem Erçen Diken, Department of Chest Diseases, Faculty of Medicine, Hitit University, Çorum, Turkey; Feza Bacakoğlu, Department of Chest Diseases, Faculty of Medicine, Ege University, Bornova, İzmir, Turkey; Süheyla Uzun, Department of Internal Medicine, Faculty of Medicine, Gaziosmanpaşa University, Tokat, Turkey; İskender Aksoy, Department of Emergency Medicine, Faculty of Medicine, Ondokuz Mayıs University, Samsun, Turkey; Hakan Cinemre, Department of Internal Medicine, Faculty of Medicine, Sakarya University, Sakarya, Turkey; Avşar Zerman, Division of Intensive Care Medicine, Adana City Training and Research Hospital, Adana, Turkey; Adnan Usalan, Department of Chest Diseases, Tarsus Medical Park Hospital, Mersin, Turkey; Işıl Özkoçak Turan, Department of Anesthesiology and Reanimation, Division of Intensive Care Medicine, Ankara Numune Training and Research Hospital, University of Health Sciences, Ankara, Turkey; Esra Özdemir, Department of Anesthesiology and Reanimation, İzzet Baysal State Hospital, Bolu, Turkey; Nevin Fazlıoğlu, Department of Chest Diseases, Faculty of Medicine, Namık Kemal University, Tekirdağ, Turkey; Fatma Yıldırım, Surgical Intensive Care Unit, Department of Chest Diseases, Dışkapı Yıldırım Beyazıt Training and Research Hospital, Ankara, Turkey; Ersin Günay, Department of Chest Diseases, Faculty of Medicine, Afyon Kocatepe University, Afyonkarahisar, Turkey; Nafiye Yılmaz, Department of Chest Diseases, Faculty of Medicine, Atatürk University, Erzurum, Turkey; Bilgehan Atılgan Acar, Department of Neurology, Faculty of Medicine, Sakarya University, Sakarya, Turkey; Belgin Akan, Department of Anesthesiology and Reanimation, Numune Training and Research Hospital, Ankara, Turkey; Hüseyin Arpağ, Department of Chest Diseases, Faculty of Medicine, Sütçü İmam University, Kahramanmaraş, Turkey; Cengizhan Sezgi, Department of Chest Diseases, Faculty of Medicine, Dicle University, Diyarbakır, Turkey; Atilla Can, Department of Thoracic Surgery, Konya Training and Research Hospital, Konya, Turkey; Murat Yalçınsoy, Department of Chest Diseases, Turgut Özal Medical Center, Faculty of Medicine, İnönü University, Malatya, Turkey; Selen Karaoğlanoğlu, Department of Chest Diseases, Ordu Training and Research Hospital, Ordu, Turkey; Abidin Şehitoğulları, Department of Thoracic Surgery, Faculty of Medicine, Sakarya University, Sakarya, Turkey; Sertaç Arslan, Department of Chest Diseases, Faculty of Medicine, Hitit University, Çorum, Turkey; Yusuf Aydemir, Department of Chest Diseases, Sakarya Training and Research Hospital, Sakarya University, Sakarya, Turkey; Ayperi Öztürk, Department of Chest Diseases and Interventional Pulmonology, Ankara Atatürk Chest Disease and Thoracic Surgery Training and Research Hospital, Ankara, Turkey; İclal Hocanlı, Department of Chest Diseases, Faculty of Medicine, Harran University, Şanlıurfa, Turkey; Bülent Tutluoğlu, Department of Chest Diseases, International Acıbadem Hospital, İstanbul, Turkey; Firuz Çapraz, Department of Chest Diseases, Marmaris State Hospital, Muğla, Turkey; Musa Salmanoğlu, Department of Internal Medicine, İstanbul Sultan Abdülhamid Training and Research Hospital, University of Health Sciences, İstanbul, Turkey; Aydanur Ekici, Department of Chest Diseases, Faculty of Medicine, Kırıkkale University, Kırıkkale, Turkey; Naci Murat, Department of Industrial Engineering, Faculty of Engineering, Ondokuz Mayıs University, Samsun, Turkey; Hatice Şahin, Department of Chest Diseases, Necip Fazıl City Hospital, Kahramanmaraş, Turkey; Sena Ataman, Department of Chest Diseases, İzmir Dr Suat Seren Thoracic Diseases and Surgery Training and Research Hospital, İzmir, Turkey; Özlem Edipoğlu, Department of Chest Diseases, Division of Intensive Care Medicine, İzmir Dr Suat Seren Thoracic Diseases and Surgery Training and Research Hospital, İzmir, Turkey; Tekin Yıldız, Department of Chest Diseases, Bursa Yüksek İhtisas Training and Research Hospital, University of Health Sciences, Bursa, Turkey; Zahide Doğanay, Department of Anesthesiology and Reanimation, Samsun Training and Research Hospital, Samsun, Turkey; Celalettin Dağlı, Department of Anesthesiology and Reanimation, Medicana Faculty of Medicine, Karatay University, Konya, Turkey; Esra Arslan Aksu, Department of Chest Diseases, Samsun Training and Research Hospital, Samsun, Turkey; Burçak Zitouni, Department of Chest Diseases, Faculty of Medicine, Karabük University, Karabük, Turkey; Ayşe İlksen Eğilmez, Department of Anesthesiology and Reanimation, Konya Training and Research Hospital, Konya, Turkey; Yeliz Şahiner, Department of Anesthesiology and Reanimation, Faculty of Medicine, Hitit University, Çorum, Turkey; Pervin Korkmaz Ekren, Department of Chest Diseases, Faculty of Medicine, Ege University, Bornova, İzmir, Turkey; Zerrin Gürel Durmuş, Department of Chest Diseases, Faculty of Medicine, Karadeniz Technical University, Trabzon, Turkey; Handan İnönü Köseoğlu, Department of Chest Diseases, Faculty of Medicine, Gaziosmanpaşa University, Tokat, Turkey; Ahmet Baydın, Department of Emergency Medicine, Faculty of Medicine, Ondokuz Mayıs University, Samsun, Turkey; Ahmet Nalbant, Department of Internal Medicine, Faculty of Medicine, Sakarya University, Sakarya, Turkey; Davut Aydın, Department of Anesthesiology and Reanimation, Division of Intensive Care Medicine, Faculty of Medicine, Ondokuz Mayıs University, Samsun, Turkey; Ahmet Bindal, Department of Anesthesiology and Reanimation, Division of Intensive Care Medicine, Ankara Numune Training and Research Hospital, University of Health Sciences, Ankara, Turkey; Şener Balas, Surgical Intensive Care Unit, Department of Chest Diseases, Dışkapı Yıldırım Beyazıt Training and Research Hospital, Ankara, Turkey; Şule Esen Karamişe, Department of Chest Diseases, Faculty of Medicine, Afyon Kocatepe University, Afyonkarahisar, Turkey; Ömer Araz, Department of Chest Diseases, Faculty of Medicine, Atatürk University, Erzurum, Turkey; Türkan Acar, Department of Neurology, Faculty of Medicine, Sakarya University, Sakarya, Turkey; Hasan Kahraman, Department of Chest Diseases, Faculty of Medicine, Sütçü İmam University, Kahramanmaraş, Turkey; Melike Demir, Department of Chest Diseases, Faculty of Medicine, Dicle University, Diyarbakır, Turkey; Cengiz Burnik, Department of Thoracic Surgery, Konya Training and Research Hospital, Konya, Turkey; Ebru Çanakçı, Department of Anesthesiology and Reanimation, Ordu Training and Research Hospital, Ordu, Turkey; Cahit Bilgin, Department of Chest Diseases, Faculty of Medicine, Sakarya University, Sakarya, Turkey; Özgür Yağan, Department of Anesthesiology and Reanimation, Faculty of Medicine, Hitit University, Çorum, Turkey; Semih Aydemir, Department of Anesthesiology and Reanimation, Atatürk Pulmonology Training and Research Hospital, Ankara, Turkey; Bülent Güçyetmez, Department of Anesthesiology and Reanimation, International Acıbadem Hospital, İstanbul, Turkey; Mine Özgün Benli, Department of Anesthesiology and Reanimation, Marmaris State Hospital, Muğla, Turkey; Yalçın Önem, Department of Internal Medicine, İstanbul Sultan Abdülhamid Training and Research Hospital, University of Health Sciences, İstanbul, Turkey.

## Authors’ contributions

Tevfik Özlü and Mehtap Pehlivanlar Küçük personally reviewed the efficacy data, understand the statistical methods employed for efficacy analysis, and confirm an understanding of this analysis, that the methods are clearly described, and that they are a fair way to report the results. Tevfik Özlü and Mehtap Pehlivanlar Küçük personally reviewed the safety data. They understand the statistical methods employed for safety analysis and confirm that they understand this analysis, that the methods are clearly described, and that they are a fair way to report the results. Tevfik Özlü and Mehtap Pehlivanlar Küçük confirm that the study objectives and procedures are honestly disclosed. Moreover, they reviewed the study execution data and confirm that procedures were followed to an extent that convinces all authors that the results are valid and generalizable to a population similar to that enrolled in this study.

Mehtap Pehlivanlar Küçük had full access to all of the data in the study and takes responsibility for the integrity of the data and the accuracy of the data analysis. 

All authors contributed substantially to the development of the study design, data analysis and interpretation, and the writing of the manuscript by participating in researchers’ meetings held online during the study. All authors enrolled their own institution’s patients for the study.

All authors read and approved the final version of the manuscript.
